# The effect of photographic light brightness on cup to disc ratio grading

**DOI:** 10.1186/s12886-021-02209-6

**Published:** 2021-12-13

**Authors:** Matthew J. McSoley, Eldar Rosenfeld, Alana Grajewski, Ta Chen Chang

**Affiliations:** 1 Bascom Palmer Eye Institute, University of Miami Miller School of Medicine, 900 NW 17th Street, Miami, FL 33136 USA; 2 Department of Ophthalmology, Tel Aviv Sourasky Medical Center, Affiliated to the Sackler Faculty of Medicine, Tel Aviv University, 6 Weizmann Street, 64239 Tel Aviv, Israel

**Keywords:** Digital fundus photography, Photographic Light Brightness, Glaucoma, Cup-to-disc ratio, Quality Control

## Abstract

**Background:**

Digital optic disc photographs are integral to remote telehealth ophthalmology, yet no quality control standards exist for the brightness setting of the images. This study evaluated the relationship between brightness setting and cup/disc ratio (c/d) grading among glaucoma specialists.

**Methods:**

Optic disc photographs obtained during routine examinations under anesthesia were collected to construct an image library. For each optic disc, photographs were obtained at 3 light intensity settings: dark, medium, and bright. From the image library, photograph triads (dark, medium and bright) of 50 eyes (50 patients) were used to construct the study set. Nine glaucoma specialists evaluated the c/d of the study set photographs in randomized order. The relationships between the brightness levels and the c/d grading as well as graders’ years in practice and variability were evaluated.

**Results:**

The c/d were graded as significantly larger in bright photographs when compared to photographs taken at the medium light intensity (0.53 vs 0.48, *P* < 0.001) as well as those taken at the dark setting (0.47, *P* < 0.001). In addition, no relationship was found between ophthalmologists’ years in practice and the variability of their c/d grading (*P* = 0.76).

**Conclusion:**

Image brightness affects c/d grading of nonstereoscopic disc photographs. The brighter intensity is associated with larger c/d grading. Photograph brightness may be an important factor to consider when evaluating digital disc photographs.

## Background

The use of color disc photography as a glaucoma screening tool was first championed by Lichter et al. in 1976 [1], and several subsequent studies comparing color disc photography to clinical examination has shown photographs to have excellent specificity and modest sensitivity in detecting glaucoma [2]. The 2019 SARS-CoV-2 pandemic (COVID-19) has galvanized renewed interest and efforts for telehealth ophthalmologic care. Nonstereoscopic disc photography is an imaging modality that is directly accessible to the patients by adapting smart phone devices [3], and thus are integral in remote telehealth ophthalmology care [4]. In particular, digital optic disc images will be central to remote glaucoma screening, evaluation, and management [5]. Currently, there are no standardized quality control protocols for obtaining digital optic disc images, with the light intensity setting being one of the variables that can change based on the device and the photographer. The effect of this variability on the assessment of the optic disc configurations is unknown [6]. In this study, we evaluated the effect of light brightness setting on c/d grading of nonstereoscopic disc images among glaucoma specialists with varying levels of experience.

## Methods

### Image library

**Fig. 1:**
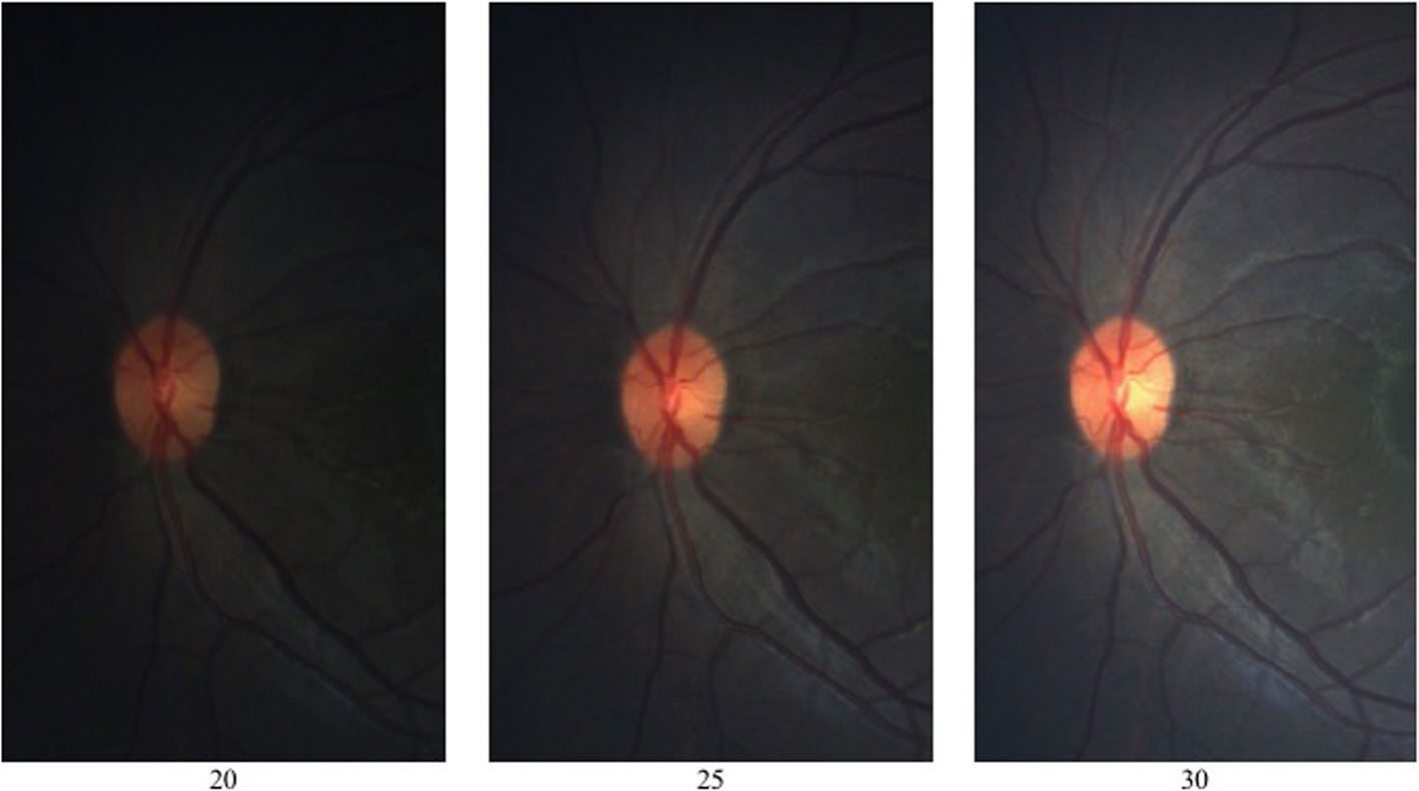
Example photographs of the same eye taken at the dark (20), medium (25), and bright (30) light intensity settings used in the study

An image library was constructed retrospectively using nonstereoscopic digital optic disc photographs obtained from pediatric patients during examinations under anesthesia using the RetCam (Natus Medical, Inc. Pleasanton, CA, USA). These patients all have unilateral or bilateral ocular pathologies, including childhood glaucoma. Each optic disc (healthy and otherwise) was imaged at 3 different brightness settings: dark (RetCam light brightness setting 20), medium (brightness setting 25), and bright (brightness setting 30), with the ambient light of the room kept the same for each capture. From this library, the images from 50 eyes of 50 children were included in the study, with 3 photographs per eye at dark, medium and bright brightness levels (total 150 separate images). Only photograph triads with clearly visible third-order vessels were included. Prior to the examinations under anesthesia, informed consent was provided by all parents or legal guardians to have any images obtained used for research and educational purposes. Example images of the same eye taken at each of the three light brightness settings are show in Fig. 1.

### Cup-to-disc ratio grading

The set of 150 photographs were presented to 9 fellowship-trained practicing glaucoma specialists on one of two high-definition, nonstereoscopic computer view screens in randomized orders. The view screens were calibrated to have same background brightness, and the ambient lighting was kept constant during the grading process. There was no time limit to completing the grading task, although the entire task was performed in one sitting without breaks. The 9 glaucoma specialists had practice range between 3 and 42 years (median 6 years). The glaucoma specialists were asked to provide a c/d estimate to the nearest 0.05.

### Data analysis

In order to assess intergrader reliability, an intraclass correlation coefficient (ICC) was calculated for each of the 3 brightness groups. ICC values range from 0 to 1, with 0 indicating no agreement between graders and 1 representing perfect agreement between graders. Commonly cited ranges for ICCs are < 0.40 indicating poor agreement; 0.40 to 0.59 indicating fair agreement; 0.60 to 0.74 indicating good agreement; and > 0.75 indicating excellent agreement between graders [7]. Mean c/d gradings for each of the 3 light settings were compared with each of the others using a paired *t* test. A paired t-test was performed in lieu of both Analysis of Variance (ANOVA) and independent samples t-test because each of the three comparisons is independently relevant, and the same 50 eyes were used for each of the three light brightness groups.

Additionally, the relationship between graders’ years in practice and variability of their c/d grades was assessed using a Pearson correlation coefficient. Coefficient of variation, defined as the standard deviation divided by the mean, was used to quantify the variability of the c/d gradings. Coefficient of variation was used in lieu of standard deviation (SD) or variance because it displays variability in the context of the mean. Calculation of the ICC values was performed using SPSS (IBM SPSS Statistics for Windows, Version 27.0); all other data analyses were performed using Microsoft Excel Version 16.30. A *P* value of 0.05 or less was considered to be statistically significant.

## Results

ICC values calculated for the dark, medium, and bright groups were 0.74, 0.71 and 0.65, respectively. Each of these indicates “good” agreement between graders and thus satisfactory intergrader reliability. Across all of the photographs evaluated by the 9 glaucoma specialists, the mean c/d was 0.50. The mean c/d (± SD) for each of the three groups was 0.47 (± 0.11) in the dark group (*n* = 50), 0.48 (± 0.11) in the medium group (n = 50), and 0.53 (± 0.12) in the bright group (n = 50).

When the 3 photograph groups were compared against each other, the bright photograph c/d gradings were statistically significantly larger compared to the medium photographs (0.53 vs 0.48, *P* < 0.001) as well as the dark photographs (0.47, *P* < 0.001) (Table 1). Although the mean c/d grading at the medium intensity (0.48) was larger than that of the dark intensity photographs (0.47), this difference is minor and fails to reach any statistical significance (Table 1).

**Table 1:** Cup to disc ratio means and comparisons among the 3 brightness settings

Mean c/d ± SD	Dark (*n* = 50)	Medium (*n* = 50)	Bright (*n* = 50)
	0.47 ± 0.11	0.48 ± 0.11	0.53 ± 0.12
	Dark vs. Medium	Dark vs. Bright	Medium vs. Bright
*P* value*	0.3030	< 0.0001	< 0.0001

The mean c/d gradings for each of the 3 brightness groups, dark, medium, and bright, were compared to each of the others using paired *t* tests*; c/d (cup to disc ratio), SD (standard deviation)

**Fig. 2:**
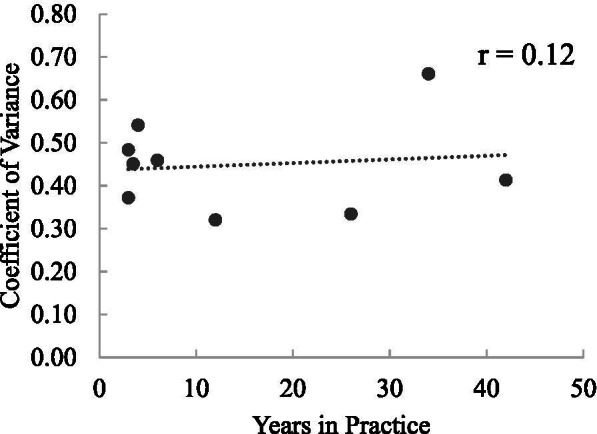
No correlation was observed between the graders’ years of experience and the variability of the c/d provided for each of the photographs

The mean c/d of the nine individual graders ranged from 0.39 to 0.56. No relationship was found between years in practice and the variability of the c/d grading (r = 0.12, *P* = 0.76) (Fig. 2).

## Discussion

Our data suggest that the light intensity used in digital nonstereoscopic color disc photography can affect the c/d estimation by glaucoma specialists. Among the same patients at the same time points, graders were more likely to assign a higher c/d if the photograph was taken at a bright intensity setting. This may have significant implications in remote telehealth glaucoma screening given the high specificity of disc photographs compared to clinical examination as a glaucoma screening tool [2, 8, 9].

For established patients, inconsistency in the light intensity setting may result in over-calling or the under-calling of a patient’s glaucoma progression over time. Additionally, discrepancies could arise if different providers caring for the same patient are using different light settings.

These results also suggest that the outcomes of glaucoma screenings may be significantly different based on the light intensity setting of the fundus camera being used. For example, in a retrospective cross-sectional study of Haitian Afro-Caribbean patients screened for glaucoma at community health fairs in Miami, FL, patients were classified as glaucoma suspects if their c/d exceeded 0.7 [8]. Within our sample, 18% of subjects would be classified as glaucoma suspects using the dark intensity setting, and 26% would be considered glaucoma suspects using the bright intensity setting. Although the two study populations differ, this illustrates that light intensity levels could potentially impact glaucoma screening outcomes. Since c/d remains a useful measure for glaucoma detection in community screening programs and retrospective studies, a standard protocol for light intensity setting may improve the accuracy of both.

This study has several limitations. First, we did not standardize the way by which the glaucoma specialists were asked the evaluate c/d nor did we assess intraobserver reliability, which may have contributed to the variability. However, this unstructured approach may simulate the real-world approach more closely than a structured c/d grading, as practitioners of remote telehealth ophthalmology likely do not utilize any fixed, standardized approach in c/d estimates either. Second, we did not measure the pupil diameter or the precise light intensity at each photograph, which limits our ability to comment on the technical details of each photograph. We also did not label and analyze separately glaucomatous vs nonglaucomatous optic discs, and thus we do not know if the effect of light brightness on c/d grading affects one type of optic discs more than the other. Lastly, our graders consisted only of glaucoma specialists, which may limit the generalizability of our data to the real world practitioners which may comprise of ophthalmologists with various training background. Nevertheless, our findings suggest that light intensity during color disc photography may be an important factor affecting c/d grading.

## Conclusions

Light intensity used during digital disc photography can significantly affect c/d estimates, which may have real world implications as telehealth ophthalmology care and remote digital disc photography become more popular. Going forward, practitioners should incorporate photograph brightness into their assessment of disc photographs, especially in assessing c/d in the setting of glaucoma screening.

## Data Availability

The data generated and analyzed during this study are available from the corresponding author on reasonable request.

## Abbreviations

c/d: Cup to disc ratio
ICC: Intraclass correlation coefficient
SD: Standard deviation
ANOVA: Analysis of Variance
